# Visual sources and information usefulness in the second-hand market: User-generated, firm-generated, and mixed photos across price conditions

**DOI:** 10.1371/journal.pone.0356763

**Published:** 2026-08-21

**Authors:** Xin Cao, Aqilah Yaacob, Kim Mee Chong

**Affiliations:** 1 School of Management and Marketing, Taylor’s Business School, Taylor’s University, Subang Jaya, Selangor, Malaysia; 2 School of Accounting and Finance, Taylor’s Business School, Taylor’s University, Subang Jaya, Selangor, Malaysia; Gannon University, UNITED STATES OF AMERICA

## Abstract

This study examines how photo source configurations are associated with perceived information usefulness in second-hand e-commerce and whether these relationships vary across displayed-price conditions. Drawing on the Information Adoption Model (IAM) and Signaling Theory, the study conceptualizes product photos as both visual evidence about product condition and seller-related market signals. A 3 × 2 between-subjects experiment was conducted using simulated iPhone product pages on Xianyu (n = 660). The experiment compared User-Generated Photos (UGPs), Firm-Generated Photos (FGPs), and Mixed Photos across higher and lower displayed-price conditions. Data were analyzed using partial least squares structural equation modeling, multi-group analysis, and supplementary interaction tests. The results show that both information quality and source credibility were positively associated with information usefulness. At the overall photo-source level, the information quality–information usefulness relationship was stronger for Mixed Photos and FGPs than for UGPs, whereas the source credibility–information usefulness relationship was strongest for FGPs. Differences across displayed-price conditions were limited and configuration-specific. The information quality relationship was stronger under the higher displayed-price condition, with the clearest difference observed for Mixed Photos, whereas no consistent price-related difference emerged for the source credibility relationship. These findings extend the application of IAM to visual information evaluation in second-hand markets by showing that photo source configurations correspond to distinct evaluative routes and that these relationships can vary across displayed-price contexts.

## Introduction

The ubiquity of smartphones has shifted consumer evaluations from text-dominant to visual-centric formats [1,2]. Photographic information is intuitive, information-rich, and cognitively efficient [3,4], making visual signals increasingly central in online purchase environments. Recent research has examined how non-numerical signals shape consumer preference formation [5], yet most empirical evidence remains concentrated on primary product markets where product quality is relatively standardized [6–10]. Second-hand e-commerce represents a more uncertain information environment because used products differ in condition, authenticity, usage history, and functional integrity. To examine visual information evaluation in this context, this study focuses on Xianyu, China’s leading C2C second-hand platform [11]. Xianyu contains heterogeneous visual sources, ranging from structured, high-clarity Firm-Generated Photos (FGPs) used by professional sellers to raw, experiential User-Generated Photos (UGPs) uploaded by individual consumers. This setting allows the study to examine how different photo source configurations shape consumers’ evaluations of used products.

Recent studies in adjacent digital decision contexts show that consumers evaluate online information through content, source, and decision stakes. In travel decisions, credible and practical user-generated content guides information seeking when consumers evaluate experiences that cannot be fully inspected before consumption [12]. In financial decision-making, the qualities of knowledge influencers affect investment willingness, with risk tolerance shaping responses to source cues [13]. In collectible markets, scarcity-related cues and fear of missing out influence willingness to pay when product value is uncertain, symbolic, and difficult to standardize [14]. Second-hand e-commerce brings these issues together in a visual product evaluation setting. Like travel decisions, it involves pre-purchase inspection uncertainty. Like financial decisions, it involves economic exposure. Like collectible markets, it involves product non-standardization. Product photos therefore become central because they show visible product condition and reveal how sellers choose to disclose privately held information about that condition [15–19].

Visual disclosure provides the conceptual link between the Information Adoption Model (IAM) and Signaling Theory. In IAM, consumers assess whether information is useful through information quality and source credibility [20–22]. In second-hand markets, these two judgments are shaped by the seller’s visual disclosure under information asymmetry. Buyers evaluate what photos reveal about product condition and what the seller’s disclosure pattern implies about source reliability. Signaling Theory explains why these judgments matter: sellers hold private information about product condition, so buyers rely on observable cues to reduce adverse selection concerns [23–25]. The seller’s control over what is photographed, how clearly it is presented, and how much evidence is disclosed determines whether visual information becomes useful through the information quality route or the source credibility route. Signaling Theory explains how observable visual disclosure shapes consumers’ judgments of information quality and source credibility, while IAM explains how these judgments contribute to perceived information usefulness.

UGPs, FGPs, and Mixed Photos provide different signal profiles. UGPs support condition-related evaluation by showing products in ordinary usage contexts [26–29]. FGPs invite credibility-related inferences when standardized presentation is interpreted as seller effort, disclosure control, and visual organization, although seller-controlled presentation can also raise concerns about selective display [27,30,31]. Mixed Photos combine experiential and standardized evidence, giving buyers a broader basis for comparing product condition and seller disclosure across source formats [32–34]. This logic complicates the common UGP–FGP assumption that user-generated content is more credible because it appears less commercially motivated. In second-hand e-commerce, credibility judgment depends on source independence, seller disclosure, hidden-defect concerns, and the completeness of visual evidence. Photo source configuration is therefore expected to create heterogeneity in the information quality and source credibility routes.

Displayed price level provides the transaction context in which consumers evaluate visual information. In information-asymmetric markets, listed price is an observable attribute that can influence the importance consumers attach to product-related information [23,24,35]. A higher displayed price represents a larger nominal transaction amount, under which the completeness and reliability of available visual evidence may become more consequential to product evaluation [36–39]. Accordingly, this study examines whether the relationships between information quality, source credibility, and information usefulness vary across higher- and lower displayed-price conditions.

Based on this logic, this study asks three research questions:

RQ1: How do information quality and source credibility contribute to perceived information usefulness in visually presented second-hand e-commerce environments?

RQ2: How do the effects of information quality and source credibility on perceived information usefulness vary across different photo source configurations?

RQ3: How do the relationships between information quality, source credibility, and information usefulness vary across higher- and lower displayed-price conditions?

This study makes three contributions. First, it clarifies how information quality and source credibility operate when consumers evaluate visual information in second-hand e-commerce. By anchoring these evaluations in visual disclosure under adverse selection, the study explains why product photos in resale markets function as both product evidence and seller-related signals. Second, it compares UGPs, FGPs, and Mixed Photos to show how photo source configuration corresponds to different evaluation routes in the formation of information usefulness. Third, it examines whether these route-specific relationships vary across higher- and lower displayed-price conditions. Together, the study provides a route-specific account of visual information evaluation in resale markets by showing how photo source configurations correspond to differences in IAM paths and how these patterns vary across displayed-price contexts.

## Theoretical development

### Visual information adoption in second-hand e-commerce

In the second-hand market, consumers face pronounced information asymmetry because the actual condition, usage history, and functional integrity of a used product cannot be directly verified before purchase [15]. This uncertainty increases the importance of observable product evidence. Photographs are particularly relevant in this setting because they allow buyers to inspect visible condition cues, compare product attributes, and reduce reliance on textual claims alone [10,16–18].

In this context, photos function as visual disclosure. They show which aspects of the product the seller chooses to reveal, how clearly these aspects are presented, and whether the listing provides enough evidence to support condition inference. Prior studies show that visual cues can reduce uncertainty [17], complement textual information [18], and strengthen perceived informativeness in online product presentation [19]. In resale markets, visual evidence is particularly consequential because incomplete disclosure and product uncertainty can expose buyers to adverse selection and financially consequential decisions [40].

The Information Adoption Model (IAM) explains how information affects consumer judgment through two key antecedents of information usefulness: information quality and source credibility [20–22]. In visual second-hand transactions, information quality refers to the extent to which product photos provide relevant, sufficient, and adequately detailed information for assessing item condition [18]. Source credibility refers to the extent to which the visual source and presentation make the seller or information provider appear knowledgeable, careful, and trustworthy [41]. Product photos become useful when they help consumers evaluate the item and reduce uncertainty about the listing. Clearer and more complete visual evidence is therefore expected to increase perceived information usefulness by improving product assessment. More credible visual sources are also expected to increase perceived information usefulness by reducing doubts about information reliability [20,42–46].

Accordingly:

H1a: At the overall level, information quality has a significant positive effect on information usefulness.
H1b: At the overall level, source credibility has a significant positive effect on information usefulness.

### Visual signal configuration and differential effects

Photo source configuration shapes how buyers evaluate visual disclosure in second-hand listings. UGPs, FGPs, and Mixed Photos differ in the type of evidence they provide, the degree of seller control they imply, and the uncertainty they address. These differences provide the basis for expecting the information quality and source credibility routes to vary across photo source configurations.

#### The role of information quality.

Information quality is closely tied to condition diagnosis in second-hand e-commerce. UGPs provide real-use evidence by showing products in ordinary usage contexts, including traces of wear, handling, and everyday presentation [4,28,29]. FGPs provide standardized visual evidence through greater clarity, consistency, and visual control [30,31]. Each single-source format has limits: UGPs can reveal authentic use conditions but often vary in angle, lighting, coverage, and completeness [47], while FGPs improve clarity and comparability but may not fully capture ordinary use conditions [48].

Mixed Photos combine these two forms of evidence. By presenting both experiential and standardized visual cues, they allow buyers to compare condition-related information across source formats and judge whether the listing provides a sufficiently complete view of the product [15,32]. This cross-source comparison strengthens the information quality route by giving buyers a broader basis for assessing product condition.

Accordingly:

H2a: The effect of information quality on information usefulness differs significantly across photo source configurations, with the expected order being Mixed > FGPs > UGPs.

#### The role of source credibility.

Source credibility refers to the extent to which the information source is perceived as knowledgeable and trustworthy [41]. In many online contexts, user-generated content is treated as more credible than firm-generated content because it appears less commercially motivated and more independent from seller persuasion [48,49]. This conventional credibility logic emphasizes source independence.

Second-hand e-commerce adds another credibility problem. Buyers evaluate whether a seller who holds private condition information has disclosed the product in a way that reduces concerns about hidden defects, incomplete coverage, and selective presentation. Credibility evaluation in resale markets therefore involves two competing heuristic routes.

The first route is the Verification Heuristic. Through this route, buyers compare different forms of visual evidence to judge whether the listing provides consistent and cross-checkable condition information. This logic favors Mixed Photos because they combine authenticity-oriented UGPs with the clarity and completeness of FGPs [32–34]. The second route is the Accountability Heuristic. Through this route, buyers infer source credibility from the seller’s visual organization, disclosure effort, and apparent responsibility for the presented product condition. This logic gives FGPs a competing credibility advantage because professionally structured photos can make the seller appear more knowledgeable, organized, and accountable for the disclosed condition of the product [30,31,50,51]. Seller-controlled presentation can also make selective display more salient, which keeps cross-source verification theoretically relevant.

H2b follows the verification side of this credibility tension. Mixed Photos are predicted to provide the strongest credibility basis because they combine source authenticity with standardized disclosure and allow buyers to verify seller-controlled presentation against ordinary-use visual evidence. UGPs are predicted to rank second because their ordinary-use presentation is less commercially controlled than FGPs. FGPs are predicted to rank third under the conventional source-independence logic because seller-controlled presentation makes selective display more salient.

Accordingly:

H2b: The effect of source credibility on information usefulness differs significantly across photo source configurations, with the expected order being Mixed > UGPs > FGPs.

### Displayed price conditions and route-specific evaluation

Displayed price level defines the transaction context in which consumers evaluate visual information. In second-hand markets, a higher listed price represents a larger monetary commitment and makes errors in product evaluation more consequential [23,24,35–39]. The importance attached to product evidence and seller-related signals may therefore vary across higher- and lower displayed-price conditions. These differences can be examined through the Verification Heuristic and the Accountability Heuristic developed above.

For the information quality route, the Verification Heuristic emphasizes the comparison and cross-checking of visual evidence. Mixed Photos combine ordinary-use evidence from UGPs with the clarity and standardized presentation of FGPs, giving buyers a broader basis for evaluating product condition [32,52]. FGPs provide structured and visually consistent evidence, while UGPs provide experiential evidence with less standardized coverage. When the displayed transaction amount is higher, the value of complete and cross-checkable evidence is expected to become more pronounced. Mixed Photos should therefore produce the strongest information quality relationship, followed by FGPs and UGPs.

Accordingly:

H3a: Under the higher displayed-price condition, Mixed Photos exert the strongest effect on the relationship between information quality and information usefulness, followed by FGPs and UGPs; under the lower displayed-price condition, these differences are not significant.

For the source credibility route, the Verification Heuristic and the Accountability Heuristic provide competing predictions. The Verification Heuristic favors Mixed Photos because the combination of ordinary-use and standardized evidence allows buyers to compare different forms of seller disclosure and assess their consistency [32–34]. The Accountability Heuristic favors FGPs because visual organization, clarity, and presentation consistency can support inferences about seller effort, expertise, and responsibility for the disclosed product condition [30,31,50,51]. UGPs retain an advantage in source independence because their presentation appears less commercially controlled.

H3b follows the Verification Heuristic. When the displayed transaction amount is higher, cross-source comparison is expected to provide the strongest basis for evaluating source credibility. Mixed Photos are therefore predicted to produce the strongest source credibility relationship, followed by UGPs and FGPs. The Accountability Heuristic provides a competing explanation under which FGPs may gain credibility from structured and professional visual disclosure.

Accordingly:

H3b: Under the higher displayed-price condition, Mixed Photos exert the strongest effect on the relationship between source credibility and information usefulness, followed by UGPs and FGPs; under the lower displayed-price condition, these differences are not significant.

**Fig 1:**
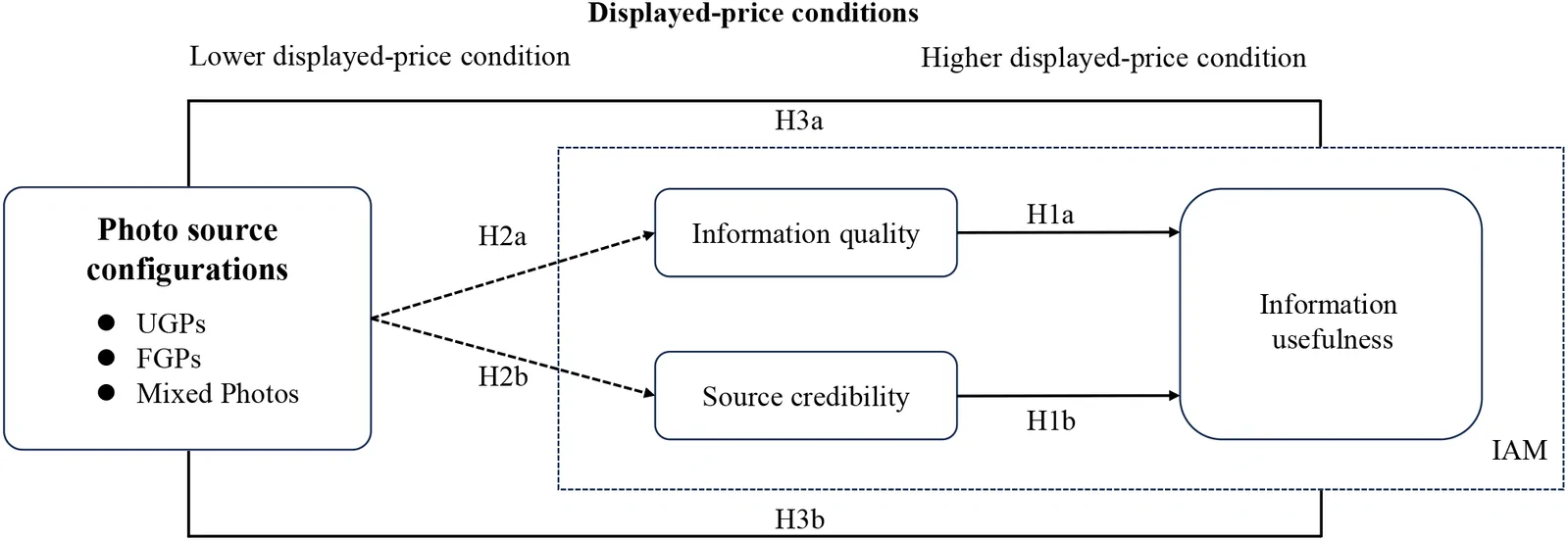
Research framework.

The research framework is presented in Fig 1.

## Method

### Research design and stimuli

This study employed a 3 (photo source configuration: UGPs, FGPs, Mixed) × 2 (displayed-price condition: higher vs. lower) between-subjects experimental design. Apple’s iPhone was selected as the focal product because it is highly active in second-hand electronics transactions and its heterogeneous functional condition makes visual cues important for reducing consumer uncertainty [53]. Apart from the assigned photo source configuration and displayed price, the page layout and basic product specifications were held constant across conditions.

Price level was operationalized using two displayed prices. The higher displayed-price condition was set at RMB 3,500, and the lower displayed-price condition was set at RMB 800. The values were selected during stimulus development with reference to national monthly disposable income statistics [54] and created an absolute difference of RMB 2,700, with the higher price being 4.375 times the lower price. Across the six experimental conditions, the page layout and basic product specifications were held constant, while the displayed price and photo source configuration varied according to the assigned condition. The two price points created clearly differentiated displayed transaction amounts, enabling comparison of the two IAM paths across higher- and lower displayed-price conditions.

The visual stimuli consisted of six simulated product pages featuring 12 standardized photos, including six UGPs and six FGPs sourced from actual listings. All textual descriptions were removed so that participants’ evaluations were based on the visual cues provided.

### Participants and procedure

Data were collected through a 36-day online survey distributed to respondents with second-hand platform experience. The survey invitation stated that the study concerned second-hand product evaluation and was intended for participants with prior Xianyu purchase experience and basic familiarity with iPhone products. At the beginning of the questionnaire, respondents reported whether they had completed at least one purchase on Xianyu within the past 12 months and whether they had basic familiarity with the focal product category. These two items were specified in advance as eligibility criteria for inclusion in the final analytical sample.

The 681 submitted questionnaires represent all completed responses recorded before the application of eligibility and data-quality filters. After data export, responses were screened using predefined criteria. Responses were removed if respondents did not meet the eligibility criteria, showed straight-lining across scale items, or completed the questionnaire in less than 60 seconds. Of the 681 submitted questionnaires, 7 were excluded due to ineligibility, 9 due to straight-lining, and 5 due to completion time below 60 seconds, yielding a final sample of 660. The final sample profile is reported in S1 Table in S1 File. Data were collected from 10 July to 15 August 2025. Ethical approval was granted by the Human Ethics Committee of Taylor’s University on 7 July 2025 (Approval No. HEC 2025/241). All participants provided electronic informed consent before beginning the questionnaire.

### Measures

All constructs were operationalized using validated multi-item scales adapted from existing literature. Source credibility and information usefulness were measured using five-item scales derived from Islam et al. [55] and Erkan and Evans [56]. Information quality was initially measured using a six-item scale adapted from prior information-adoption research [22], covering the relevance, sufficiency, quantity, credibility, factual basis, and evaluative contribution of the photographic information. All items used a 7-point Likert scale. The full measurement items and references are listed in S2 Table in S1 File.

### Data analysis

The measurement and structural models were assessed using partial least squares structural equation modeling in SmartPLS 4 (version 4.1.0.9). Bootstrapping with 5,000 resamples was used to test the significance of the structural paths. Measurement invariance was assessed using the MICOM procedure before multi-group comparisons were conducted across photo source and displayed-price conditions. Supplementary interaction analyses were conducted in Python 3.13.5 using statsmodels 0.14.6 and ordinary least squares regression with HC3 heteroskedasticity-robust standard errors. Construct scores were calculated as the means of the indicators retained in the final measurement model. Price was coded as 0 for the lower displayed-price condition and 1 for the higher displayed-price condition. The supplementary regressions included a pooled displayed-price interaction model, separate models for each photo source configuration, an FGPs–UGPs slope comparison, and pairwise photo-source slope comparisons within each displayed-price condition. All statistical tests were two-tailed, with statistical significance evaluated at α = 0.05.

## Results

### Measurement model assessment and diagnostic tests

Following Hair et al. [57], the measurement model was refined through a joint assessment of indicator loadings, construct boundaries, and content coverage. Six items were removed: IQ1, IQ3, IQ6, IU1, IU2, and SC5. Each removed item showed inadequate empirical performance and was also examined in relation to the conceptual meaning of its construct.

For information quality, IQ1 used credibility-related wording that overlapped with the source credibility construct, while IQ3 required respondents to judge whether photographic information was fact-based, a judgment that could not be independently verified from the visual stimuli. IQ6 described an improvement in product understanding and was therefore closer to an outcome of information use than to an attribute of information quality. The retained information quality items captured relevance, sufficiency and detail, and adequacy of information quantity. For information usefulness, IU1 provided a broad global evaluation that was represented more specifically by the retained indicators, while IU2 used the term “informative,” which overlapped conceptually with information quality. The retained indicators captured task efficiency, ease of making an appropriate choice, and the overall advantage of using photographic information. For source credibility, SC5 assessed honesty, a meaning already represented by the retained credibility, trustworthiness, and reliability indicators. SC1–SC4 continued to cover both source experience and trustworthiness-related judgments. The refinement therefore reduced cross-construct overlap while preserving the core conceptual content of all three constructs.

After refinement, all constructs satisfied the recommended thresholds (Cronbach’s α and Composite reliability > 0.70; AVE > 0.50). Information quality achieved an AVE of 0.714 (α = 0.800; CR = 0.882), source credibility reached an AVE of 0.676 (α = 0.840; CR = 0.893), and information usefulness yielded an AVE of 0.750 (α = 0.833; CR = 0.900), indicating satisfactory internal consistency and convergent validity (see S3 Table in S1 File). Discriminant validity was confirmed through the Fornell–Larcker criterion and HTMT ratios (see S4 Table in S1 File) [58].

Common method bias (CMB) was addressed through experimental design and statistical diagnostics. The two experimental factors, photo source configuration and price level, were exogenously assigned rather than self-reported by respondents, which structurally separated the manipulated grouping variables from the perceptual outcome measures. Full collinearity VIF values ranged from 1.102 to 1.265, below the 3.3 threshold [59]. As a preliminary traditional diagnostic, Harman’s single-factor test showed that the first component explained 26.29% of the variance, below the 50% reference threshold [60]. Together, the exogenous experimental manipulation and full collinearity results indicate that common method variance was unlikely to substantially distort the estimated relationships. Path-level VIFs were approximately 1.103, indicating low multicollinearity in the structural model [61]. Detailed diagnostic results are provided in S5 Table in S1 File.

### Structural model and hypothesis testing

#### Overall path analysis.

Before the multi-group analysis, measurement invariance was assessed using the MICOM procedure. Compositional invariance was established for all constructs in the FGPs–Mixed, UGPs–Mixed, and displayed-price comparisons. In the FGPs–UGPs comparison, compositional invariance was established for information quality and information usefulness (see S6 Table in S1 File) [62]. Structural paths were then tested using bootstrapping with 5,000 resamples. As shown in Table 1, information quality had a significant positive effect on information usefulness (β = 0.271, t = 7.222, p < 0.001), supporting H1a. Source credibility also had a significant positive effect on information usefulness (β = 0.371, t = 11.026, p < 0.001), supporting H1b. These results show that both visual information quality and visual source credibility contributed to perceived information usefulness in the second-hand product evaluation context.

**Table 1:** Summary of path analysis for H1a and H1b.

	Original sample (O)	Sample mean (M)	Standard deviation (STDEV)	T statistics (|O/STDEV|)	P values
**Information quality to information usefulness**	0.271	0.272	0.037	7.222	p < 0.001
**Source credibility to information usefulness**	0.371	0.372	0.034	11.026	p < 0.001

#### Multi-group analysis (MGA) across photo source configurations.

The MGA examined whether the two IAM paths differed across Mixed Photos, FGPs, and UGPs. For the information quality path, Mixed Photos (β = 0.401, p < 0.001) and FGPs (β = 0.337, p < 0.001) were significant, whereas the path was not statistically significant for UGPs (β = 0.097, p = 0.145). Pairwise comparisons showed that Mixed Photos significantly exceeded UGPs (difference = 0.304, p = 0.001), and FGPs also exceeded UGPs (difference = 0.239, p = 0.005). Mixed Photos and FGPs did not differ significantly (p = 0.421). H2a was supported for the Mixed Photos–UGPs and FGPs–UGPs comparisons, whereas the predicted difference between Mixed Photos and FGPs was not supported.

For the source credibility path, all three within-group coefficients were significant. FGPs produced the largest coefficient (β = 0.561, p < 0.001) and significantly exceeded Mixed Photos (β = 0.304, p < 0.001; difference = 0.257, p < 0.001). UGPs also showed a significant source credibility relationship (β = 0.249, p < 0.001), while Mixed Photos and UGPs did not differ significantly (difference = 0.054, p = 0.526). Because compositional invariance was not established for source credibility in the FGPs–UGPs comparison, this contrast is reported using the supplementary HC3 slope analysis. The HC3 estimate showed a stronger source credibility slope for FGPs than for UGPs (b = 0.344, 95% CI [0.158, 0.530], p < 0.001; see S8 Table in S1 File). H2b was not supported.

These findings are summarized in Table 2, with detailed pairwise MGA results reported in S7 Table in S1 File and supplementary HC3 results reported in S8 Table in S1 File.

**Table 2:** Multi-group path coefficients across photo source configurations.

Path	Mixed (β)	FGPs (β)	UGPs (β)
Information quality to information usefulness	0.401***	0.337***	0.097
Source credibility to information usefulness	0.304***	0.561***	0.249***

Notes: β values represent standardized path coefficients within each subgroup. Significance markers are based on within-group path tests. * p < 0.05, ** p < 0.01, *** p < 0.001.

#### Differences across displayed-price conditions.

To examine whether the two IAM paths differed directly between the higher- and lower displayed-price conditions, supplementary regression analyses were conducted using the retained construct-item means and heteroskedasticity-robust standard errors. Price was coded as 0 for the lower displayed-price condition and 1 for the higher displayed-price condition, with photo source configuration included as a categorical control. The interaction between information quality and displayed price was positive and significant (b = 0.165, 95% CI [0.012, 0.318], p = 0.035). By contrast, the interaction between source credibility and displayed price was not significant (b = 0.029, 95% CI [−0.132, 0.191], p = 0.721).

Further analyses were conducted separately for each photo source configuration. The information quality × price interaction was significant for Mixed Photos (b = 0.314, 95% CI [0.061, 0.567], p = 0.015), but not for FGPs (b = −0.003, p = 0.983) or UGPs (b = 0.214, p = 0.119). None of the source credibility × price interactions were statistically significant. The information quality × displayed-price interaction was significant in the Mixed Photos subgroup, whereas the corresponding interactions in the FGP and UGP subgroups were not statistically significant.

The MGA then compared photo source configurations within each displayed-price condition. Under the higher displayed-price condition, the information quality path was strongest for Mixed Photos (β = 0.563, p < 0.001), followed by FGPs (β = 0.309, p < 0.001) and UGPs (β = 0.184, p = 0.028). Pairwise comparisons showed that Mixed Photos significantly exceeded FGPs (difference = 0.254, p = 0.013) and UGPs (difference = 0.378, p = 0.001). The difference between FGPs and UGPs was not significant (p = 0.230). The predicted advantage of Mixed Photos was therefore supported, but the full ordering of Mixed Photos > FGPs > UGPs was not established.

Under the lower displayed-price condition, the information quality path was significant for Mixed Photos (β = 0.277, p = 0.001) and FGPs (β = 0.381, p < 0.001), but not for UGPs (β = 0.052, p = 0.743). The PLS-MGA showed a significant difference between FGPs and UGPs (difference = 0.329, p = 0.023). The supplementary HC3 slope comparisons also showed significant FGPs–UGPs and Mixed Photos–UGPs differences, while Mixed Photos and FGPs did not differ significantly (see S8 Table in S1 File). The predicted absence of photo-source differences under the lower displayed-price condition was not supported. Overall, H3a was supported for the predicted advantage of Mixed Photos under the higher displayed-price condition, whereas the full ordering and the predicted absence of lower-price differences were not supported.

For the source credibility path, the higher-price results were contrary to the predicted ordering. FGPs produced the strongest effect (β = 0.631, p < 0.001) and significantly exceeded both Mixed Photos (β = 0.297, p < 0.001; difference = 0.334, p < 0.001) and UGPs (β = 0.217, p = 0.092; difference = 0.414, p < 0.001). Mixed Photos and UGPs did not differ significantly (p = 0.567). Under the lower displayed-price condition, the source credibility path was significant for Mixed Photos (β = 0.296, p = 0.002), FGPs (β = 0.482, p < 0.001), and UGPs (β = 0.318, p < 0.01), but none of the pairwise differences were statistically significant. Supplementary HC3 slope comparisons produced the same higher- and lower-price pattern, and H3b was not supported (see S8 Table in S1 File).

These findings are summarized in Table 3. Detailed MGA results are reported in S7 Table in S1 File, and the supplementary interaction analyses are reported in S8 Table in S1 File.

**Table 3:** Multi-group path coefficients across photo source configurations by price level.

Price Condition	Path	Mixed (β)	FGPs (β)	UGPs (β)
Higher displayed-price condition	Information quality to information usefulness	0.563***	0.309***	0.184*
	Source credibility to information usefulness	0.297***	0.631***	0.217
Lower displayed-price condition	Information quality to information usefulness	0.277**	0.381***	0.052
	Source credibility to information usefulness	0.296**	0.482***	0.318**

Notes: β values represent standardized path coefficients within each subgroup. * p < 0.05, ** p < 0.01, *** p < 0.001.

## Discussion

The findings provide a route-specific interpretation of visual information usefulness in second-hand e-commerce. The Verification Heuristic offers a framework for understanding how buyers compare visual evidence and assess whether a listing provides a coherent basis for product evaluation. The Accountability Heuristic offers a complementary explanation for why visual organization, disclosure effort, and professional presentation may contribute to source credibility judgments. Together, these heuristics provide an interpretive framework for the different path patterns observed across photo source configurations.

The information quality results support the Verification Heuristic. At the overall photo-source level, Mixed Photos and FGPs both produced stronger information quality effects than UGPs. Under the higher displayed-price condition, Mixed Photos produced the strongest information quality effect and significantly exceeded both FGPs and UGPs. This pattern is consistent with the view that cross-source comparison contributes to visual diagnosticity in second-hand e-commerce. Mixed Photos may strengthen this route by combining standardized presentation with ordinary-use visual evidence, allowing buyers to inspect the same product through more than one visual frame [28,29,32]. In an information-asymmetric resale setting, this cross-source structure increases the diagnostic value of the listing and strengthens the path from information quality to information usefulness.

The source credibility results reveal a different theoretical logic. H2b and H3b were built on source independence and cross-source verification, leading to the prediction that Mixed Photos would generate the strongest credibility effect. The empirical results identify the limit of this prediction: FGPs produced the strongest source credibility effect overall and under the higher displayed-price condition. The Accountability Heuristic provides one explanation for this credibility reversal. Under adverse selection, professionally structured photos can make the seller appear more knowledgeable, organized, and accountable for the disclosed condition of the product. This interpretation provides a coherent theoretical account of why FGPs outperformed Mixed Photos and UGPs on the source credibility route.

This credibility reversal is one of the central theoretical findings of the study. Existing UGP–FGP research often assumes that user-generated content enjoys a credibility advantage because it appears less commercially controlled. The present results show that this assumption does not transfer cleanly to second-hand e-commerce. In resale transactions, the buyer’s credibility judgment is tied to the seller’s possession of private condition information. A professionally structured visual display can therefore function as a credibility cue when it signals seller effort in making the product inspectable. The unsupported H2b and H3b predictions are theoretically informative because the observed credibility pattern is more consistent with the Accountability Heuristic than with the Verification Heuristic.

The results across displayed-price conditions reveal a more concentrated pattern. The interaction analysis identified price-related variation in the information quality route. In the source-specific analyses, the information quality × displayed-price interaction reached significance for Mixed Photos. In this configuration, the stronger information quality relationship under the higher displayed-price condition is consistent with the Verification Heuristic. Combining standardized presentation with ordinary-use evidence provides a broader basis for assessing product condition when the listed transaction amount is higher. However, the lower displayed-price condition did not eliminate differences among photo source configurations, as FGPs still produced a significantly stronger information quality relationship than UGPs.

The source credibility route showed a different pattern. Neither the overall source credibility × price interaction nor the configuration-specific interactions were statistically significant. The strong FGP coefficient under the higher displayed-price condition therefore reflects the comparative advantage of FGPs over Mixed Photos and UGPs within that condition. This pattern is consistent with the Accountability Heuristic, under which structured and professional visual presentation supports credibility judgments about seller organization, expertise, and disclosure effort.

Taken together, the findings show that the two IAM routes respond differently to visual source configuration. Mixed Photos and FGPs provide stronger information quality relationships than UGPs, while FGPs provide the strongest source credibility relationship. This route-specific pattern explains the supported and unsupported components of H2a and H3a, together with the rejection of H2b and H3b, within a single theoretical framework.

## Conclusion

This study examined how information quality and source credibility contribute to visual information usefulness in second-hand e-commerce and how these relationships vary across photo source configurations and displayed-price conditions. Information quality and source credibility both contributed positively to information usefulness, confirming the relevance of IAM in a visual resale context. The two routes responded differently to photo source configuration. Mixed Photos and FGPs produced stronger information quality relationships than UGPs, while FGPs produced the strongest source credibility relationship.

The study develops a route-specific account of visual information evaluation under information asymmetry. Product photos function as both condition evidence and seller-related signals. The Verification Heuristic offers an explanation for why combining standardized and ordinary-use evidence supports product-condition evaluation, while the Accountability Heuristic offers a possible explanation for the source credibility advantage associated with professionally structured photographs. Displayed-price differences were limited to the information quality relationship.

The findings also support more differentiated visual strategies for second-hand platforms and sellers. The stronger information quality relationship observed for Mixed Photos supports their use in listings where buyers need to compare condition evidence across visual formats, including products with visible wear, repair histories, or uncertain functional condition. The stronger source credibility relationship observed for FGPs supports their use when visual organization and professional presentation are central to seller evaluation. Visual guidance should therefore reflect the informational demands of the product and transaction context rather than applying one photo configuration uniformly across all listings. This study has several limitations. The experiment used simulated iPhone listings on Xianyu, limiting the empirical setting to one platform and one product category. The displayed-price conditions compared RMB 3,500 and RMB 800 without a subjective manipulation check. The observed price-related differences capture variation across displayed transaction amounts, while perceived financial risk was not directly assessed. Because the product specifications were held constant, the RMB 800 condition may also have prompted concerns about product authenticity or undisclosed defects. The displayed-price factor may therefore have carried additional product-related inferences alongside differences in transaction amount. The Accountability Heuristic provides an interpretive framework for the source credibility pattern reported in the study. The measurement model covered information quality, source credibility, and information usefulness, and did not include consumers’ perceptions of seller accountability, expertise, or disclosure effort. These features define the scope within which the findings are considered.

## Supporting information

S1 File S1 Table. Demographic characteristics and background information of the participants. S2 Table. Measurement items. S3 Table. Outer loadings, reliability, and convergent validity of the refined measurement model. S4 Table. Discriminant validity results (Fornell-Larcker criterion and HTMT ratio). S5 Table. Evaluation of common method bias and multicollinearity. S6 Table. Assessment of measurement invariance (MICOM). S7 Table. Pairwise MGA results across photo source configurations and displayed-price conditions. S8 Table. Supplementary regression analyses of displayed-price and photo-source differences.
(RAR)
